# GMP development and preclinical validation of CAR-T cells targeting a lytic EBV antigen for therapy of EBV-associated malignancies

**DOI:** 10.3389/fimmu.2023.1103695

**Published:** 2023-02-02

**Authors:** Xi Zhang, Tiaoxia Wang, Xiaona Zhu, Yong Lu, Mingpeng Li, Zhihong Huang, Deping Han, Longzhen Zhang, Yang Wu, Liantao Li, Frank Klawonn, Renata Stripecke

**Affiliations:** ^1^ Biosyngen/Zelltechs Pte. Ltd., Singapore, Singapore; ^2^ Department of Radiotherapy, the Affiliated Hospital of Xuzhou Medical University, Xuzhou, China; ^3^ Biostatistics Group, Helmholtz Centre for Infection Research, Braunschweig, Germany; ^4^ Institute for Information Engineering, Ostfalia University, Wolfenbuettel, Germany; ^5^ German Centre for Infection Research (DZIF), Partner Site Hannover-Braunschweig and Partner Site Cologne-Bonn, Cologne, Hannover, Germany; ^6^ Laboratory of Regenerative Immune Therapies Applied, Department of Hematology, Hemostasis, Oncology and Stem Cell Transplantation, Hannover Medical School, Hannover, Germany; ^7^ Clinic I for Internal Medicine, University Hospital Cologne, University of Cologne, Cologne, Germany; ^8^ Institute for Translational Immune-Oncology, Cancer Research Center Cologne-Essen (CCCE), University of Cologne, Cologne, Germany

**Keywords:** CAR-T cell, GMP, EBV, gp350, nasopharyngeal carcinoma, gastric carcinoma, lymphoma

## Abstract

**Introduction:**

Epstein-Barr virus (EBV) is a widely spread pathogen associated with lymphoproliferative diseases, B/ T/ NK cell lymphomas, nasopharyngeal carcinoma (NPC) and gastric carcinoma (GC). EBV lytic reactivations contribute to the genomic instability, inflammation and tumorigenesis of NPC, promoting cancer progression. Patients with NPC refractory to standard therapies show dismal survival. EBV gp350 is an envelope protein detectable in NPC specimens intracellularly and on the cell membrane of malignant cells, and is a potential viral antigen for T cell-directed immunotherapies. The potency of T cells engineered with a chimeric antigen receptor (CAR) targeting gp350 against EBV^+^ lymphoproliferative disease was previously shown.

**Methods:**

Here, we advanced towards preclinical and non-clinical developments of this virus-specific CAR-T cell immunotherapy against NPC. Different gp350CAR designs were inserted into a lentiviral vector (LV) backbone.

**Results:**

A construct expressing the scFv 7A1-anti−gp350 incorporating the CD8 transmembrane and CD28.CD3ζ signaling domain (ZT002) was selected. High titer ZT002 (~1x10^8^ TU/ml) was manufactured in HEK 293T/17 suspension cells in serum free media as large-scale production under good manufacturing practices (GMP). A LV multiplicity of infection (MOI) of 1 resulted in high frequencies of functional gp350CAR^+^ T cells (>70%) at a low (<2) vector copy numbers in the genome. ZT002 was therefore used to establish gp350CAR-T batch run production methods. GMP upscaling and validation of T cell transduction and expansion in several runs resulted in average 3x10^9^ gp350CAR-T cells per batch. >80% CD3^+^ gp350CAR-T cells bound to purified gp350 protein. *In vitro* cytotoxicity and cytokine secretion assays (IFN-γ and TNF-α) confirmed the specificity of gp350CAR-T cells against gp350^+^ NPC, GC and lymphoma cell targets. Immunocompromised B-NDG mice (NOD.CB17-*PrkdcscidIl2rgtm1*/Bcgen) were challenged s.c. with a EBV^+^ NPC C666.1 cell line expressing gp350 and then treated with escalating doses of gp350CAR-T cells or with non-transduced T cells. gp350CAR-T cells promoted antitumor responses, bio-distributed in several tissues, infiltrated in tumors and rejected gp350^+^ tumor cells.

**Discussion:**

These results support the use of gp350CAR-T cells generated with ZT002 as an Innovative New Drug to treat patients with solid and liquid EBV-associated malignancies.

## Introduction

Epstein-Barr Virus (EBV) is an ubiquitous pathogen and infects more than 95% of healthy adults. Although EBV’s primary infection is asymptomatic and mostly controlled by a potent CD8^+^ T cellular response (1), this oncogenic virus is classified as a group 1 carcinogen by the World Health Organization and it is associated with 2% of the human cancers developing worldwide (2). The primary EBV lytic infection occurs in B cells and epithelial cells homing in the buccal cavity and EBV is an important etiological factor for development of epithelial cancers such as nasopharyngeal carcinoma (NPC) (3). The persistence of episomal EBV genome and expression of several latency-associated viral proteins (LMP-1, EBNA-1) have been linked with malignant transformation in NPC (4). Notwithstanding, the contribution of EBV lytic reactivation and expression of EBV lytic products showed significant carcinogenic effects by increasing the genomic instability and tumorigenesis of NPC cells (5). NPC patients with advanced and recurrent disease have high mortality rates, and therefore targeting the EBV lytic infection may be a novel effective strategy to develop new therapies (6).

Copies of the BLLF1 gene encoding the gp350 envelope protein is detected in 25% of NPC biopsies by real-time (RT)-PCR (7). In the viral envelope, gp350 is an entry protein which is abundantly expressed during lytic reactivations and is sporadically expressed on the surface of transformed cell lines (8) (9). We showed previously that T cells transduced with different retroviral vector designs and expressing chimeric antigen receptors (CARs) targeting gp350 detected and killed EBV^+^gp350^+^ lymphoblastoid cell lines (LCLs). The best *in vitro* performing CAR-T cells incorporated the 7A1-anti-gp350 scFv, an immunoglobulin (Ig) transmembrane domain and the CD28.CD3ζ signaling domain. Nod-Rag-gamma (NRG) mice fully humanized with human CD34^+^ hematopoietic stem cells, infected with the lytic EBV-M81/fLuc strain and developing lymphoproliferative disease (LPD) demonstrated 75% therapeutic responses after CD8^+^ 7A1-gp350CAR-T cell administration (9).

In this current work, we advance towards the clinical development of gp350CAR-T cells for future immunotherapy clinical trials against NPC. We generate gp350CAR-T cells using lentiviral vectors (LVs) produced under good-manufacturing practices (GMP). We show that gp350CAR-T cells manufacturing can be up-scaled to yield sufficient cell numbers and high purity for clinical use. We demonstrate *in vitro* potency of the preclinical gp350CAR-T cells using different types of gp350^+^ tumor targets. We establish proof-of-concept of GMP-like gp350CAR-T cells used therapeutically in an EBV^+^/gp350^+^ NPC xenograft model to recognize and promote eradication of the NPC tumor *in vivo*. In sum, we confirm the applicability of gp350CAR-T cell immunotherapy against NPC.

## Materials and methods

More information on materials and methods can be found in the supplemental information.

### Ethics statements

Leukapheresis units or peripheral blood mononuclear cells were purchased from Allcells (Alameda, CA, US) or collected from donors from Shanghai Zhaxin Traditional Chinese and Western Medicine hospital (study protocol number: LP202006) with signed informed consent. All handling and care of animals were performed under the guidelines for the Care and Use of Animals for Scientific Purposes issued by the research ethics committee at Guangzhou Regenerative Medicine and Health, Guangdong Laboratory (GRMH-GDL) following the Guide for the Care and Use of Laboratory Mice (Institute of Laboratory Animal Resources, Commission on Life Sciences, National Research Council, China). Procedures used are designed to conform to accepted practices and to minimize or avoid causing pain, distress, or discomfort in the mice. In those circumstances in which the required study procedures could cause pain or distress, the mice received appropriate analgesics or anesthetics, as ascribed by the Study Director and/or the veterinary staff and approved by the Institutional Animal Care and Use Committee (IACUC) at GRMH-GDL (IACUC serial number: 2020125). When mice showed high levels or distress or reached the experimental endpoint, they were humanely euthanized with anesthesia overdose or CO_2_ inhalation. NPC tissue sections were provided by Xuzhou Medical University upon ethic committee approval (XYFY2021-KL317-02).

### Antibodies

The anti-gp350 monoclonal antibody clone 72A1 was obtained commercially (Merck Millipore, Kenilworth, NJ) and the clone 7A1 was kindly provided by Dr. Reinhard Zeidler and produced by the Core facility “Monoclonal Antibodies” at Helmholtz Zentrum Munich; **Table S1**). The mouse hybridoma cell line producing the OT6 anti-gp350 monoclonal antibody was kindly provided by Prof. Jaap Middeldorp, Amsterdam University Medical Center and the purified OT6 antibody was manufactured by Helmholtz Zentrum Munich, Germany.

### Cell lines

The EBV^+^ C666.1 human NPC cell line was purchased from Shunran Biology (Shanghai, China). Prof. Reinhard Zeidler (Ludwig-Maximilians-University Munich, Germany) kindly provided the PCI-1 human oropharyngeal cancer cell line. C666.1 and PCI-1 cells were cultured in Dulbecco’s modified Eagle’s medium (DMEM; Thermo Fisher Scientific, Waltham, MA, USA) supplemented with 10% fetal bovine serum (FBS; Nobimpex, Herbolzheim, Germany). The K562 human chronic myeloid leukemia cell line and the EBV^+^ KATO-III human gastric cancer cell line were purchased from the American Type Culture Collection (ATCC, Manassas, VA) and cultured in Iscove’s modified Dulbecco’s medium (IMDM; Thermo Fisher Scientific, Waltham, MA, USA) supplemented with 10% FBS. Jurkat cells (Clone E6-1) were obtained from ATCC and cultured in Roswell Park Memorial Institute (RPMI, Thermo Fisher Scientific, Waltham, MA, USA) + 10% FBS. 293T cells were obtained from ATCC, and cultured in DMEM + 10% FBS. All cell lines were cultured at 37^o^C and 5% CO_2_.

### Cell lines expressing gp350

To generate cell lines stably overexpressing gp350, the parental cell lines were transduced with a gp350-expressing lentiviral vector (produced by the contract research organization TransferGene Co. Ltd, Dalian, China). After expansion, gp350^+^ cells were selected after fluorescence activated cell sorting (FACS).

### Generation of EBV^+^ LCLs

The EBV^+^ B95-8 Marmoset LCL was purchased from MITO Biological technology Co. Ltd (Shanghai, China). B95-8 cells were cultured in RPMI supplemented with 10% FBS. For lytic activation and release of EBV, B95-8 cells were treated with 20 ng/ml 12-O-tetradecanoylphorbol-13-acetate (TPA). Peripheral blood mononuclear cells (PBMCs) were obtained from AllCells (Alameda, CA, USA) after approval by the ethical committee of Shanghai Zhaxin Traditional Chinese and Western Medicine hospital. After TPA activation, B95-8 cell supernatants were 1:10 diluted in RPMI + 10% FBS culture medium and used to infect PBMCs in the presence of 20 nM Tacrolimus (FK506, Aladdin, Shanghai, China). Around 20 days after infection, outgrown LCLs were identified by flow cytometry as CD23^High^CD58^+^ cells.

### Construction and production of lentiviral vectors expressing gp350CARs

The gp350CAR DNA sequences were synthesized and inserted between the BamHI and SalI sites of the pCDH-EF1-MCS-T2A-copGFP lentiviral vector (GENEWIZ, Suzhou, China). The T2A-copGFP sequences were deleted from the vector because GFP is known to be highly immunogenic (10). Batches of third generation lentiviral vectors were generated under GMP-compatible processes using proprietary methods from a contract research organization (CRO, TransferGene Co. Ltd, Dalian, China). Specifically, HEK 293T/17 cells (ATCC, Manassas, VA) first adapted to suspension cells in serum-free medium (Sino Biological, Beijing, China) by medium switching and continuous rocking at 37^o^C and cells were established and validated in Canvestbio (Wuhan, China). Transfergene (Dalian, China) manufactured and characterized high quality grade plasmids (vector backbone and three packaging plasmids) according to GMP requirements. All the plasmids were sequenced to confirm their structures. Third generation lentiviral vectors were produced after transient co-transfection of HEK 293T/17 suspension cells with four plasmids (the transfer plasmid pCDH-ZT002, the plasmid pMD2.G expressing the vesicular stomatitis virus G (VSV-G) envelope, and the packaging plasmids pMDLg/pRRE and pRSV-Rev) using a standard polyethyleneimine (PEI)-based method (PEIpro, polyplus-transfection, Illkirch, France). The cell supernatants were harvested 48 h after transfection. After pilot vector productions in flasks, the SOP for manufacturing of the LV was established and optimized (**Table S1**).

### Concentration and purification of the lentiviral vector

After filtration with 0.5 μm filters (Cobetter, Hangzhou, China), cell supernatants were treated with 40 U/ml SuperNuclease (Sino Biological, Beijing, China) for 1 h at 37^o^C. LV batches were concentrated in a hollow-fiber system (Repligen, Waltham, MA, USA) and purified with Capto Core 700 chromatography resin (Cytiva, Marlborough, MA). After filtration with 0.22 μm filters (Merck Millipore, Kenilworth, NJ), the LV batches were aliquoted in 1.8 ml samples dispensed in cryopreservation tubes (Corning, NY, USA). The LV batches were stored at -80^o^C. Vector particle concentration was determined by p24 ELISA (Takara Bio, Japan). Infective lentivirus titer was determined by virus serial dilution, infection of Jurkat cells and the percentages of CAR^+^ cells were determined by flow cytometry. Specifically, Jurkat cells were seeded in wells with 1x10^5^ cells each. Cells were transduced with 50, 10, 2, 0.4 or 0.08 μl of the LV sample. Controls were non-transduced mock cells. CAR expression was detected 72 hours post transduction by flow cytometry. gp350CAR expression was detected with APC-conjugated goat anti-human IgG-Fc directed against the IgG4 spacer (Jackson ImmunoResearch Laboratories, Philadelphia, PA, USA (**Table S1**). Activity titer was calculated using following formula: Activity titer (TU / ml) = P / V x 10^3^ × 10^5^ (P: Percentage of positive-stained cells; V: volume of cell supernatant with lentivirus used for infection).

### Production of gp350CAR-T cells

PBMCs were isolated from leukapheresis units using Ficoll density gradient separation (GE Healthcare, Chicago, IL, USA) and cryopreserved. PBMCs were thawed and cultured in AIM-V (Thermo Fisher Scientific, Waltham, MA) supplemented with 5% CTS^TM^ Immune Cell Serum Replacement (SR) (Thermo Fisher Scientific, Waltham, MA) and human IL-2 (300 IU/ml, Quangang, Shandong, China). PBMCs were activated and expanded with CTS^TM^ Dynabeads^TM^ CD3/CD28 (Thermo Fisher Scientific, Waltham, MA) for 24 h. T cells were transduced with LVs at multiplicity of infection (MOI) of 1. Three days after transduction, the cells were extensively washed to remove the LV particles. T cells were further expanded in AIM-V medium supplemented with 5% CTS^TM^ Immune Cell SR (Thermo Fisher Scientific, Waltham, MA) for three to ten days in the presence of IL-2 (300 IU/ml, Quangang). The cell product was washed twice with normal saline (Qidu pharmaceuticals, Shandong, China) containing 2.5 % human serum albumin (CSL Behring, King of Prussia, PA, USA). Cells were resuspended in Cryostor^®^ CS10 Cell Freezing Medium (STEMCELL Technologies, Vancouver, Canada) for cryopreservation and stored in liquid nitrogen.

### Quality control of gp350CAR-T cells by flow cytometry analyses

Anti-human CD3, CD4 and CD8 monoclonal antibodies were obtained from Biolegend (San Diego, CA, USA (**Table S1**)). Staining of gp350CAR was performed with the APC-conjugated goat anti-human IgG-Fc or with gp350 recombinant protein labelled with Fluorescein-5-isothiocyanate (FITC, Sino Biological, Beijing, China). Flow cytometry data was acquired and analyzed with CytoFLEX (Beckman Coulter, Brea, CA, USA).

### Analyses of secreted IFN-γ and TNF-α by ELISA

gp350CAR-T cells were co-cultured with gp350^+^ or with wild-type (w.t.) control cells at various Effector : Target (E : T) ratios for 16 h. Cell supernatants were collected for measurement of IFN-γ (Biolegend, San Diego, CA, USA) or TNF-α (BD Biosciences, Franklin Lakes, NJ, USA) by ELISA according to the manufacturers’ protocols. Samples were analyzed by microplate reader (TECAN, Männedorf, Switzerland).

### Cytotoxicity assays

After co-culture with targets at different E:T ratios for 16 h, the cytotoxic activity of gp350CAR-T cells was measured using CytoTox 96^®^ Non-Radioactive Cytotoxicity Assay (Promega, Madison, MI, USA) measuring release of lactate dehydrogenase (LDH). Alternatively, we used the DELFIA^®^ EuTDA Cytotoxicity Reagents kit (Perkin Elmer, Waltham, MA, USA) based on loading cells with a fluorescence enhancing ligand that forms a fluorescent chelate once cells are lysed. Both assays were performed strictly following the manufacturer’s instructions. Control groups were set up to measure: (i) the medium background (no cells added), (ii) the spontaneous release (target cells only), and (iii) the maximum release (target cells treated with 10 μl lysis buffer). Experiments were performed in triplicates. Data acquisition was performed using microplate reader (TECAN, Männedorf, Switzerland). For CytoTox 96^®^ Non-radioactive cytotoxicity kit, killing efficacy was calculated by using the following formula: % Cytotoxicity= [Target plus Effector (OD 490nm) – Effector Spontaneous (OD 490nm)] / [(Target maximum (OD 490nm) – Target Spontaneous (OD 490nm)] x 100. For DELFIA® EuTDA Cytotoxicity kit, killing efficacy was calculated by using the following formula: % Specific release = [Target plus Effector (counts) - Spontaneous release (counts)] / [Maximum release (counts) - Spontaneous release (counts)] x 100.

### NPC xenograft mouse model

Immunocompromised B-NDG mice (NOD.CB17-*Prkd^cscid^Il2rgt^m1^*/Bcgen) were purchased from Biocytogen (Beijing, China). A mouse xenograft model of human NPC was established by subcutaneous inoculating 5x10^6^ C666.1/gp350 cells on day 0. Five days after tumor inoculation, tumor length (L) and width (W) were measured with a caliper and the tumor volume (V) was calculated with the formula V = (L x W x W) / 2 as baseline and treatments were administered. To test the potency of gp350CAR-T cells *in vivo*, mice were randomly distributed into treatment groups: (i) i.v. injected saline (control group), (ii) i.v. injected mock non-transduced T cells (at 5x10^5^, 1x10^6^, 5x10^6^ cell doses), and (iii) i.v. injected gp350CAR-T cells (at 5x10^5^, 1x10^6^, 5x10^6^ total cell doses). Blood was collected on days 8, 19 and 27 after treatment. Plasma IFN-γ was analyzed using cytometric bead assay (BD Biosciences, Franklin Lakes, NJ, USA) strictly according to manufacturer’s protocol. Cross-sectional analyses to evaluate the bio-distribution of CAR-T cells in tissues were performed on days 6, 8, 12 and 19 and the numbers of CAR copies were measured by qPCR as described below. Tumor progression was monitored by tumor volume measurements. On day 27, mice were sacrificed and the weight of tumors was measured.

### Tissue distribution analyses of gp350CAR-T cells by PCR

Various tissues were extracted and subjected to DNA extraction using QIAamp DNA Blood Mini Kit (Qiagen, Hilden, Germany). The primer set amplifying the DNA fragment by qPCR in the gp350CAR region of the vector (forward primer: 5’- AGTTCGCTTGCGACATCTAC; reverse primer: 5’- GCCTAGACCTCTTGCTTCTATTT) was used. Gene-amplified products were detected with the probe: FAM TGCTGCTGCTGTCTCTGGTAGTC. Copies of CAR sequences were quantified in a QuantStudio 5 Real-Time PCR System (Thermo Fisher Scientific).

### Immunohistochemistry analyses of gp350^+^ and CD3^+^ cells in mouse and human tissues

The NPC tissue sections obtained from patients and used as references for gp350 IHC analyses were provided by Xuzhou Medical University, China. Tumors obtained from mice were fixed and paraffin embedded using routine methods. The OT6 monoclonal antibody (hybridoma kindly provided by Prof. Jaap Middeldorp, Amsterdam University Medical Center and antibody manufactured by Helmholtz Zentrum Munich, Germany) was used for immunohistochemical staining to identify gp350-positive cells in the tissue. The anti-CD3D & CD3E heterodimer antibody was purchased from Sino Biological, Beijing, China. Paraformaldehyde-fixed, paraffin-embedded tissues are baked for 30 minutes at 60°C and cooled to room temperature. The sections were further deparaffinized in xylene for 3 times, and gradually rehydrated with 95%, 80%, 70%, and 60% alcohol, and finally washed with distilled water twice. Endogenous peroxidase activity was quenched with a 10-minute incubation of 3% hydrogen peroxide at room temperature. Sections were further incubated in sodium citrate antigen retrieval solution (Boster, Wuhan, China) at 100^o^C boiling water bath for 10 minutes. After antigen retrieval, sections were blocked with 5% BSA at 37°C for 30 minutes, and stained with mouse-derived OT6 antibody (1:200) or anti-CD3D & CD3E heterodimer antibody (1:200, Sino Biological, Beijing, China) at 4°C overnight. HRP polymer-labeled goat anti-mouse or rabbit IgG antibody (Boster, Wuhan, China) was used as secondary antibody. Sections were further stained with the ready-to-use SABC (Boster, Wuhan, China) and DAB (Boster, Wuhan, China). Slides were further counterstained with hematoxylin (Servicebio, Wuhan, China) for 10s, then use 60%, 70%, 80%, 95% alcohol to dehydrate, and finally transparent with xylene, and mount with neutral resin (Boster, Wuhan, China). Sections were further observed under microscope (Mshot, Guangzhou, China) and captured with Mshot camera (Mshot MD50, Guangzhou, China).

### Statistical analyses

Statistical analyses were carried out with SPSS and R. Bar plots show mean ± s.d. Welch t-test with correction for Bonferroni-Holm multiple testing – if adequate – to evaluate differences in means.

## Results

### Pre-testing of LVs expressing different gp350CAR designs

**Figure 1:**
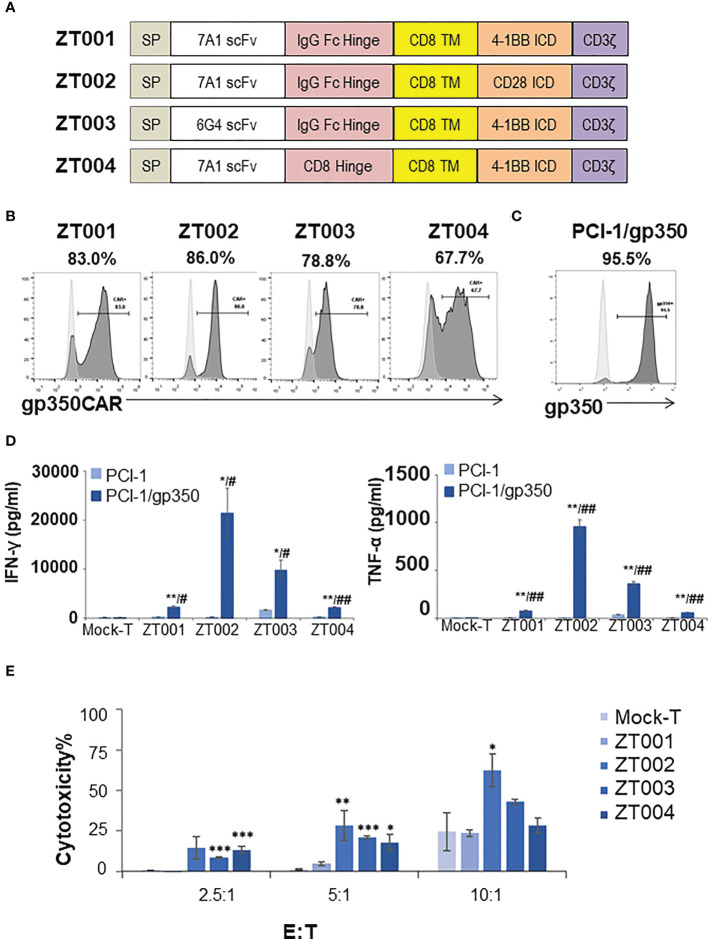
Testing of different lentiviral vectors incorporating different gp350CAR designs and selection of the best construct. **(A)** Schematic diagrams of LV constructs expressing gp350CAR. SP, signal peptide; scFv, single chain variable fragment (7A1 or 6G4); Hinge (IgG Fc or CD8); TM, transmembrane domain (all CD8); ICD, intracellular domain (CD28.CD3ζ or 4-1BB.CD3ζ). **(B)** Flow cytometry analyses showing surface expression of the CAR on T cells transduced with four lentiviral constructs (ZT001, ZT002, ZT003 or ZT004). Transduced T cells (dark grey) are compared with mock-T cells (light grey). Percentages of gp350CAR-expressing cells are shown; representative example from triplicate transduction experiments performed with T cells derived from one donor. **(C)** Flow cytometry analyses of PCI-1 cells transduced to express gp350 (dark grey) compared with un-transduced cells (light grey). **(D)** IFN-γ and TNF-α release. Mock-T and gp350 CAR-T cells were co-cultured with PCI-1 or PCI-1/gp350 cells for 16 hours at an E:T of 5:1 and the levels of IFN-γ and TNF-α released in the medium were measured by ELISA. Data are presented as means from triplicates ± SD. Welch t-test and p-values after correction for multiple comparisons, CAR-T versus Mock-T cells, *P ≤0.05, **P≤0.01, ***P ≤0.001. PCI-1/gp350 versus PCI-1, ^#^P ≤0.05, ^##^P≤0.01. **(E)** *In vitro* cytotoxicity comparison of four anti-gp350 CAR. Lactose dehydrogenase (LDH)-based cytotoxicity assay (16 hours culturing) was used to assess the cytotoxicity of four anti-gp350 CAR-T cells against gp350-positive human oropharyngeal cancer cell lines PCI-1 (PCI-1/gp350). Non-transduced T (Mock-T) cells were included as a control. These results are presented as means from triplicates ± s.d. Welch t-test and p-values after correction for multiple comparisons, compared to Mock-T, *, P < 0.05; **, P<0.01; ***, P < 0.001.

We had previously produced gp350CAR-T cells using retroviral vectors (9) but since third generation self-inactivating (SIN) LVs remains currently as the leading the gene transfer tool for CAR-T cell manufacturing (11), we designed and tested four LVs containing the CD8 transmembrane domain (TM) and incorporating the 7A1 or 6G4 gp350-specific scFvs, the human IgG Fc or CD8 hinges, and the 4-1BB.CD3ζ or CD28.CD3ζ chimeric signaling domains (**Figure 1A**). One day after activation with cytokines, PBMCs were transduced with the LVs and further expanded *ex vivo*. Analyses of the cells seven days after transduction showed variable frequencies of CAR^+^ T cells (**Figure 1B**). The cells transduced with LV-ZT002 (7A1-scFV/ Fc hinge/ CD28.CD3ζ) showed the highest frequency of gp350CAR^+^ T cells (86%, representative results from triplicate experiments, **Figure 1B**). These results confirmed previous data comparing CARs generated with retroviral vectors, showing a higher expression for vectors containing the 7A1 scFv and CD28.CD3ζ (9). In order to compare them functionally, gp350CAR-T cell types produced with the four different LVs were co-cultured with human oropharyngeal cancer cell line PCI-1 engineered to express gp350 (PCI-1/gp350, results from triplicate experiments, **Figure 1C**). PCI w.t. cells were used as control targets and the release of IFN-γ and TNF-α were measured by ELISA. Secretion of cytokines was significantly higher for all types of gp350CAR-T cells co-cultured with gp350^+^ cells than the control co-cultures (using Mock effectors or targeting PCI w.t.), confirming specific gp350 target recognition for all gp350CAR designs (**Figure 1D**). gp350CAR-T cells generated with the LV-ZT002 produced approximately tenfold higher cytokine levels than gp350CAR-T cells generated with the vector ZT001 incorporating the 4-1BB co-stimulatory domain (**Figure 1D**). gp350CAR-T cells generated with the vector ZT003 (6G4-gp350CAR-Fc-41BB.CD3ζ) or with the LV ZT004 (7A1-gp350CAR-CD8-41BB.CD3ζ) also showed much lower cytokine levels when stimulated with cells expressing gp350 than the CAR-T cells transduced with LV-ZT002. The *in vitro* killing activities of the different gp350CAR-T cells were analyzed by cytotoxicity assays performed as independent triplicates (**Figure 1E**). The CAR-T cells generated with the vector ZT002 showed the highest killing capacity of PCI/gp350^+^ target cells, and up to approximately 70% cytotoxicity at E:T ratio of 10:1. The experiment was repeated using a range of E:T ratios 1:2, 1:1, 2:1, 4:1 and the superior cytotoxicity activity of CAR-T cells generated with the ZT002 vector was confirmed (**Figure S1**). These data showed that gp350CAR-T cells incorporating the 7A1-scFV and CD28.CD3ζ elements showed the highest expression and functionality, and the vector ZT002 was chosen for further clinical development.

### LV design, GMP production, purification and testing

**Figure 2:**
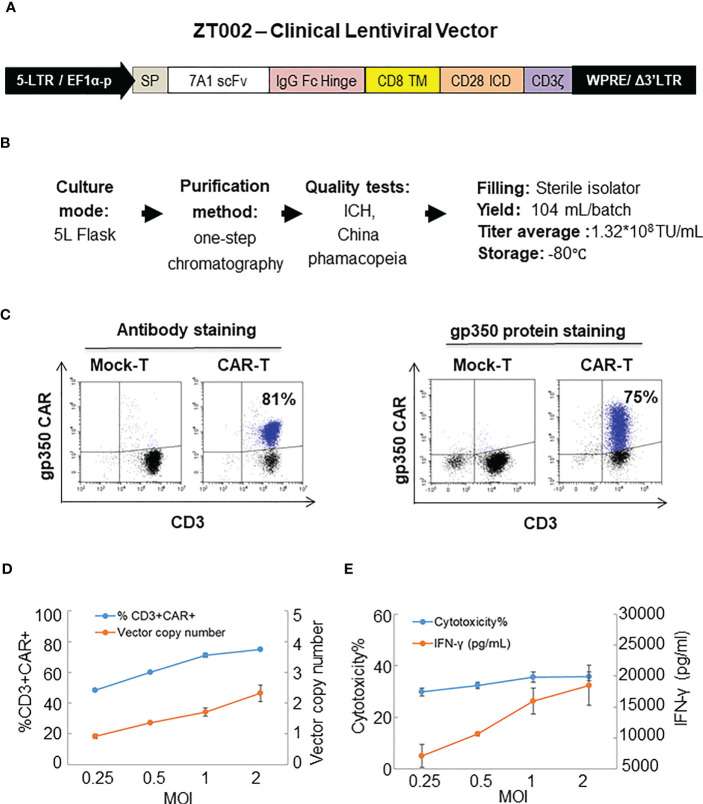
Production of ZT002 using GMP-compliant methods and generation of gp350CAR-T cells. **(A)** Detailed vector scheme of the self-inactivating third generation ZT002 vector selected for clinical use. The vector contains non-homologous 5’ and 3’ long-terminal repeats (LTRs). The 5’ LTR incorporates the EF1-α promoter and a WPRE element to improve the RNA stability upstream of the mutated (Δ) 3’ LTR. **(B)** Scheme of LV-ZT002 production, purification, testing and average results for three productions. **(C)** Flow cytometry detection of gp350CAR using an antibody for detection of the hinge (anti-IgG Fc, left) or labelled recombinant gp350 protein binding to the scFv (right). **(D)** Testing different transduction conditions with increasing multiplicity of infection (MOI) to correlate the efficiencies of gp350CAR^+^ T cell generation with the integrated vector copy numbers. PBMCs were stimulated on day 0, transduced on the next day with ZT002 at increasing MOIs (0.25, 0.5, 1 and 2), and ten days later analyzed for the percentages of CD3^+^CAR^+^ cells (blue line) and number of CAR copies per cell genome (orange line). **(E)** Testing the functionality of gp350CAR-T cells generated with different ZT002 MOIs for target cytotoxicity and correlation with IFN-γ production. CAR-T cells were co-cultured with PCI-1/gp350 (E:T=2.5:1) and IFN- γ released in medium (orange line) and cytotoxicity activity (blue line) were analyzed. Results of independent triplicate experiments, performed with gp350CAR-T cells produced with one PBMC donor.

The ZT002 construct was used for LV scale-up production and purification (see main regulatory elements of the self-inactivating (SIN) vector in **Figure 2A**). GMP-compliant methods were established for upscaling, purification, filing and quality control (QC) of the vector product (**Figure 2B**). The validation of manufacturing of three independently produced consecutive batches LV-ZT002 by analyses of titers showed highly reproducible results (**Table 1**). All different production methods and scales, ranging from 50 ml flasks to the 5 l bioreactor system, resulted in satisfactory lentivirus yield (**Table S2**). Infection of Jurkat with the crude virus or with the purified/concentrated virus showed in average titers of 2.85 x 10^7^ transduction units (TU) and 1.32 x 10^7^ TU, respectively. The final volume of approximately 100 ml of virus product per batch showed an average of 15% LV recovery after purification. The quality tests were performed according to the “International Council for Harmonization of Technical Requirements for Pharmaceuticals for Human Use” (ICH) (https://www.ich.org/) and the Chinese pharmacopeia (http://wp.chp.org.cn/front/chpint/en/). Detailed release criteria are listed in **Table S3**.

**Table 1:** Validation of LV-ZT002 GMP manufacturing after production and testing of three independent and consecutive viral lots.

Batch Number	Total volume before purification (ml)	Titer crude supernatant Jurkat cells (TU/ml x10^7^)	Total volume after purification (ml)	Filling volume per vial (ml)	Activity titer Jurkat cells (TU/ml x10^8^)	Recovery rate (%)
LV-ZT002-20210403	3,600	2.47	104	0.3	1.37	16.01
LV-ZT002-20210404	3,447	2.42	99	0.3	1.11	13.16
LV-ZT002-20210405	3,478	2.85	110	0.3	1.48	16.43
Average ± SD	3,508 ± 81	2.85 ± 0.24	104 ± 6	0.3	1.32 ± 0.19	15.20 ± 1.78

### Up-scaled batch runs of gp350CAR-T cells

The GMP-grade clinical scale ZT002 vector was then used for upscaling production of CAR-T cells. Initially, we transduced T cells at different LV multiplicities of infection (MOIs; 0.25, 0.5, 1, 2). After transduction, T cells expanded approximately 100-fold for nine days. The identity analyses was performed by flow cytometry using a fluorochrome-labelled antibody recognizing the IgG Fc-Hinge region in the CAR or with a fluorochrome-labeled gp350 protein binding to the scFv (**Figure 2C**), confirming that the gp350CAR-T cells could bind the target protein. We observed a correlation between the transduction efficiency (measured by detection of CAR^+^ CD3^+^ T cells by flow cytometry) and the number of integrated vector copies in the genome (**Figure 2D**). We also observed that CAR-T cell produced with higher LV MOIs secreted higher levels of IFN-γ when incubated with PCI-1/gp350 target cells (**Figure 2E**). However, their cytotoxic activities reached the plateau at MOI = 1 (**Figure 2E**). According to “points to consider of quality control test and pre-clinical study for CAR-T cell therapy products” by the National Institutes for Food and Drug Control, a low vector copy number (VCN) (less than five copies per cell) is required to minimize the potential risks of insertional mutagenesis. Since the MOI of 1 resulted in high frequencies of functional gp350CAR^+^ T cells (>70%) at a low (<2) CAR copy numbers, the MOI of 1 was chosen for gp350CAR-T batch run productions methods (**Table 2**). The recovery of T cells after expansion for ten days was in average 3.81 x 10^9^ cells. >80% CD3^+^ gp350CAR^+^ T cells (at similar CD4^+^CAR^+^ and CD8^+^CAR^+^ ratios) were detected after staining with immunoconjugated gp350 protein and FACS analyses (**Table 2**). In average, 2.37 VCN per genome were detectable by real-time qPCR (**Table 2**). Based on the test-runs of gp350CAR-T cell productions, several relevant and required parameters for the batch release criteria were determined (**Table S4**). Therefore, the scale-up methods for generation of GMP-like gp350CAR-T cells were straightforward and resulted in CAR-T cells with the expected high expansion and viability, high purity, low vector copy numbers, and no detectable contaminations (bacteria and mycoplasma, **Table S4**).

**Table 2:** Validation of gp350CAR-T cell manufacturing after production and testing of three independent and consecutive batch runs.

Batch Run	Expansion-fold day 10	Total cells (x10^9^)	% CD3^+^ CAR^+^	% CD4^+^	% CD8^+^	LV copies/ cell
1	205	2.77	83.4	51.3	45.4	2.10
2	334	5.01	86.5	38.9	55.2	1.91
3	405	3.65	82.1	44.6	52.8	3.10
Average ± SD	315 ±101	3.81 ±1.13	84.0 ±2.3	44.9 ±6.2	51.1 ±5.1	2.37 ±0.64

### *In vitro* potency testing of GMP-like gp350CAR-T cells

**Figure 3:**
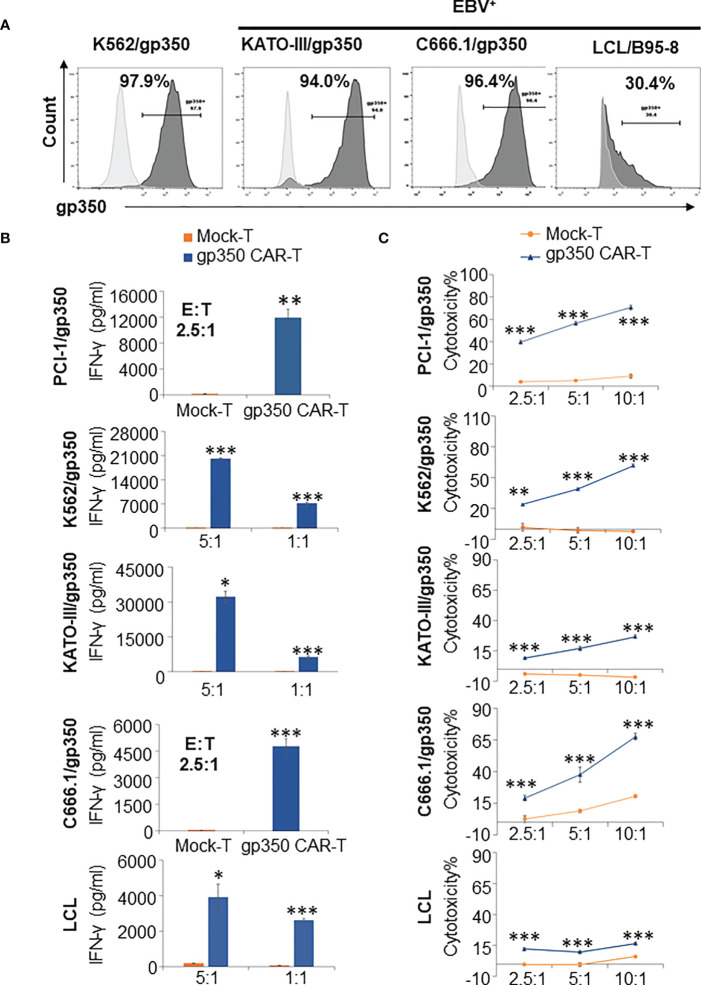
Potency testing of gp350CAR-T cells produced after scale-up against different cell targets expressing gp350. **(A)** Flow cytometry analyses of gp350 expression on various cancer cell lines: EBV^-^ PCI-1 expressing gp350 (PCI-1/gp350), EBV^-^ myeloid leukemia line K562 expressing gp350 (K562/gp350), EBV^-^ gastric carcinoma KATO-III expressing gp350 (KATO-III/gp350), EBV^+^ nasopharyngeal carcinoma C666.1 expressing gp350 (C666.1/gp350), and an EBV^+^ B95-8-derived lymphoblastoid cell line (LCL). **(B)** Analyses of secreted IFN-γ. Gp350CAR-T cells were co cultured with above tumor cell lines for 16 h at indicated E:T ratios and IFN-γ release level was quantified by ELISA. t test, compared to mock-T, *P≤0.05, **P≤0.01, ***P≤0.001. **(C)** *In vitro* cytotoxicity assays. gp350CAR-T were co-cultured with PCI-1/gp350, KATO-III/gp350, C666.1/gp350 and LCLs and LDH-based cytotoxicity assay (performed after 16 h of co-culture) or Delfia EuTDA cytotoxicity assay (performed after 2 h of co-culture) were performed. Mock-T cells were included as controls. The results are presented as means ± s.d. t test, compared to mock-T, *P≤0.05, **P≤0.01, ***P≤0.001. Independent triplicate experiments were performed with gp350CAR-T cells produced with one PBMC donor.

When NPC primary samples are immortalized to generate NPC lines, they commonly lose the expression of lytic antigens after extended culture. Therefore, we generated lentivirus-transduced gp350^+^ cell lines: EBV^-^ PCI-oropharyngeal carcinoma 1 (as shown in **Figure 2**), EBV^-^ K562 myeloid leukemia, EBV^-^ GC KATO-III, EBV^+^ NPC C666.1. In addition, we obtained a LCL transformed and immortalized line expressing gp350 after *in vitro* infection of B cells with B95-8 EBV. The gp350-overexpressing cell lines showed >90% stable gp350 expression, and the EBV-transformed LCLs showed ~30% gp350 expression (**Figure 3A**). gp350CAR-T cells were co-cultured with those cell lines at variable E:T ratios for 16 h. Culture supernatants were harvested for IFN-γ ELISA measurements. For all co-cultures, gp350CAR-T cells showed significantly higher concentrations of secreted IFN-γ (100~3,000 fold) than control mock-T cells (**Figure 3B**). *In vitro* killing efficacy of gp350CAR-T cells was evaluated in triplicate experiments with various E:T ratios after 16 h of co-culture with the cell targets. For all E:T ratios used, gp350CAR-T cells showed significantly higher cytotoxicity against all cell targets than control mock-T cells (**Figure 3C**). Therefore, we confirmed the specificity, reactivity and cytotoxicity of gp350CAR-T cells produced after scale-up methods.

### gp350CAR-T cells significantly reduce EBV^+^ NPC C666.1/gp350 tumor burden *in vivo*

**Figure 4:**
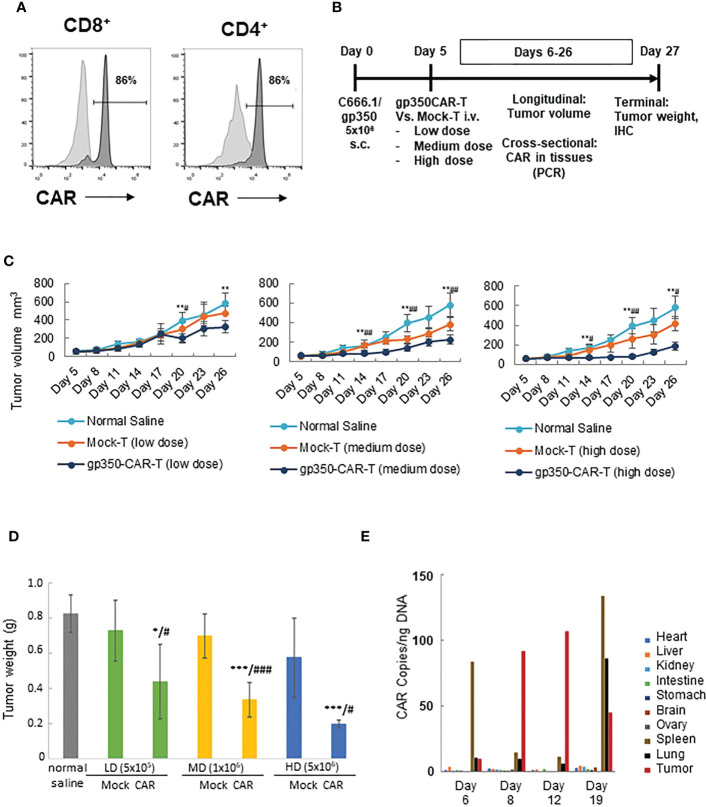
*In vivo* testing of gp350CAR-T cells in B-NDG mice challenged with NPC. **(A)** Flow cytometry analyses of GMP-like gp350CAR-T cells used for *in vivo* experiments showing high frequencies of gp350CAR^+^ CD8^+^ and CD4^+^ T cells. **(B)** Schematic representation of *in vivo* experiment. Mice were injected with 5x10^6^ NPC EBV^+^C666.1/gp350 cells s.c. Five days later, mice were injected with saline, mock-T or CAR-T cells at escalating cell doses. Longitudinal, cross-sectional and terminal analyses were performed to follow tumor growth, CAR-T cell bio-distribution and therapeutic specificity. **(C)** Longitudinal analyses of tumor volume (mm^3^). Left graph: low T cell dose (5x10^5^); middle graph: medium T cell dose (1x10^6^); right graph: high T cell dose (5x10^6^). **(D)** Terminal analyses of tumor weight (g). Grey: saline control; green: low T cell dose; yellow: medium T cell dose; blue: high T cell dose. **(E)** Cross-sectional analyses for detection of CAR sequences in tissues (qPCR; copies/ng). Welch t-test and p-values after correction for multiple comparisons, compared to saline control group, *P<0.05; **P<0.01; ***P<0.001; compared to Mock-T, ^#^P<0.05; ^##^P<0.01; ^###^P<0.001.

A batch of gp350CAR-T cells produced with the GMP-like methods and showing 86% CD8^+^CAR^+^ and 86% CD4^+^CAR^+^ cells (**Figure 4A**) was used for *in vivo* testing. Since subcutaneous (s.c.) implantation of the NPC cell line C666-1 cells into immune deficient mice consistently engraft and produce tumors (12), we used the C666-1/gp350 cells to establish a xenograft mouse model. C666.1/gp350 cells were injected s.c. on the flanks of B-NDG mice, and five days later we measured the baseline volume of the tumors (**Figure 4B**). On day five after challenge, the mice were then randomized into seven groups (n=6 mice per cohort) and injected with (i) Saline control, (ii) low dose (5x10^5^/mouse) mock-T or (ii) gp350CAR-T cells, (iv) medium dose (1x10^6^/mouse) mock-T or (v) gp350CAR-T cells, an (vi) high dose (5x10^6^/mouse) mock-T or (vii) gp350CAR-T cells. The tumor volumes were measured longitudinally and mice were sacrificed at day 27 after tumor implantation for collection of biopsies and terminal analyses (**Figure 4B**). The tumor volumes were measured longitudinally (n=6) until day 26 after implantation. For all T cell doses tested, until day 26, gp350CAR-T cells promoted a dose-dependent and significant reduction of the tumor volumes compared with mock-T cells (**Figure 4C**). Accordingly, administration of gp350CAR-T cells at all different doses promoted a significant reduction of tumor weight measured at day 27 compared with mice injected with mock-T cells or saline (**Figure 4D**). gp350CAR-T cells were also injected at the highest 5x10^6^ cell dose into B-NDG mice (n=2) five days after tumor implantation for a cross-sectional analyses of CAR-T cells bio-distribution. The mice were sacrificed at days 6, 8, 12 and 19 after tumor challenge for analyses. CAR copies were analysed by qPCR (CAR copies/ ng of tissue DNA) (**Figure 4E**). One day after gp350CAR-T cell infusion, CAR sequences were mostly detectable in spleen. From three to twelve days after CAR-T cell injections, CAR sequences were mostly noticeable in tumors. At day nineteen after CAR-T injections, the CAR PCR signal was widely distributed in tumors, spleen and lungs and in minor degrees in several other tissues. Hence, gp350CAR-T cells showed a dose-dependent therapeutic activity against gp350^+^NPC tumor growth and CAR sequences were detectable in the tumor and afterwards redistributed systemically.

### gp350 expression is decreased in C666.1/gp350 NPC tumors after therapy with gp350CAR-T cells

**Figure 5:**
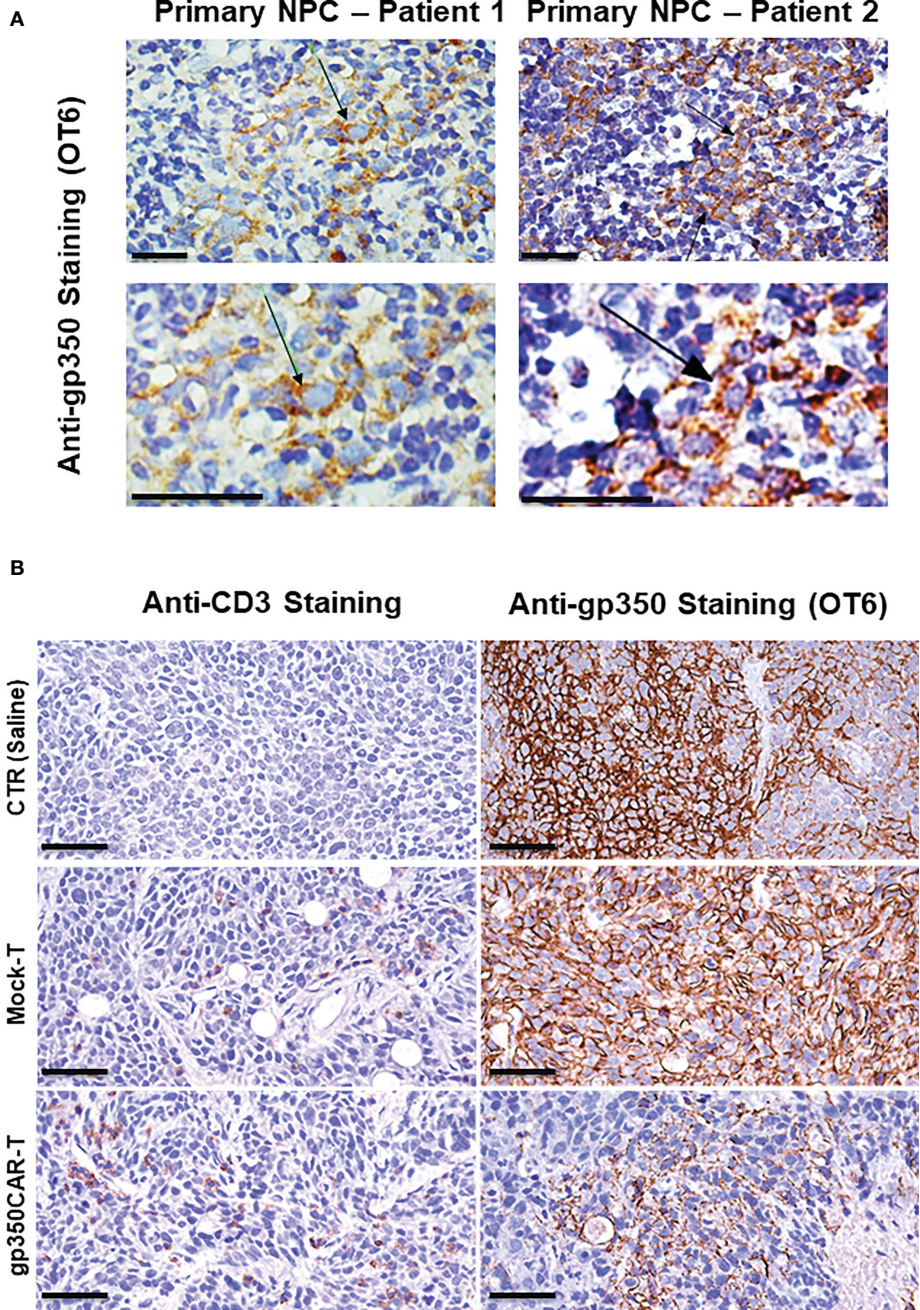
Immunohistochemistry staining of paraffin-embedded sections obtained from primary NPC patient tissues or obtained from a C666.1/gp350 tumor sample explanted from a mouse. **(A)** anti-gp350 OT6 staining on primary tumor biopsies from two NPC patients. Arrows, positive staining on tumor cell membrane. Scale bar, 50 μm. **(B)** Representative example of anti-human CD3 staining and anti-gp350 staining on C666.1/gp350 tumor samples. Additional two cases can be found in **Figure S1**. Scale bars represent 50 μm.

NPC biopsies obtained from patients occasionally express gp350 and we confirmed this using the OT6 mouse antibody for IHC analyses, showing strong gp350-positive staining in scattered cell populations (**Figure 5A** left panels, representative example). Applying the same staining methods, gp350 was detectable in the tumors of mice challenged with C666.1/gp350 tumors (**Figure 5A** right panels, representative example). This confirmed that our *in vivo* model reflected recent findings obtained with new tumor xenografts that could be established with EBV-positive NPC (13). We therefore evaluated if there was a correlation between gp350CAR-T cells homing the tumors and loss of gp350^+^ cells (**Figure 5B**, **Figure S2**, **Table S5**). Whereas control mice treated with saline or with mock-T cells showed no or very little T cells homing in the tumor, mice treated with gp350CAR-T cells showed distinguishable single or clustered CD3^+^ T cells infiltrating the tumor (**Figure 5B** left panels, representative example, **Figure S2** with two additional cases, **Table S5**). IHC analyses for gp350 detection demonstrated that infiltration of gp350CAR-T cells in C666.1/gp350 tumors was inversely correlated with detection of gp350-positive cells (**Figure 5B** right panels, representative example, **Figure S2** with two additional cases), indicating a causal effect for tumor eradication. An expert pathologist provided a semi-quantitative microscopic assessment of cell frequencies and expression levels of CD3 and gp350 in C666.1/gp350 tumor tissue sections obtained from mice treated with saline, Mock-T cells or gp350CAR-T cells. Cells with strong and moderate staining levels were quantified separately. Despite the limitations of the semi-quantitative assessment, mice infused with gp350CAR-T cells showed in average lower gp350 expression in tumors and higher frequencies of CD3^+^ cells than mock T cells (**Table S5**).

After completion of the preclinical experiments, we hired a contract research organization to conduct non-clinical experiments using good laboratory practices (GLP) and documentation for the submission of an Investigational New Drug (IND) for initialisation of clinical trials. Pharmacodynamics studies confirmed the immunotherapeutic activity of gp350CAR-T cells against C666.1/gp350 tumors at a dose dependent manner (**Figure S3**). The mice treated with gp350CAR-T cells in the non-clinical model did not show weight loss, signs of cytokine release syndrome or neurotoxicity. On the other hand, mice treated with mock-T cells showed pronounced weight loss, which was clinically defined by the CRO as Graft-versus-Host Disease (GvHD), which is a result of the xenograft reactivity of the human T cells against the mouse tissues. In addition, a pharmacokinetics study was performed under GLP to evaluate the bio-distribution of the test article in female and male mice (**Figure S4**). The biodistribution of the CAR-T cells was monitored by cross-sectional analyses by PCR analyses one day after administration and up to day 70 of the experiment. For both sexes, gp350CAR-T cell detection was initially mostly detected in tumors until day 21. From day 49 until 70, gp350CAR-T cells were also frankly detectable in blood, spleen and liver (**Figure S4**). Although gp350CAR-T cells were also detected in the spinal cord and several other tissues from day 49 onwards, mice did not show signs of cytokine release syndrome or neurotoxicity until the endpoint.

## Discussion

After pioneering clinical trials by June et al showing impressive clinical responses, CAR-T cells have since then revolutionized the field of tumor immunotherapy, especially in the immunotherapy of refractory hematological tumors (14, 15). CAR-T cell therapies have produced sensational long-term remissions for approximately 40% of patients with multiply relapsed/refractory aggressive B-cell non-Hodgkin lymphomas, resulting in several approved advanced cell therapy products (14). However, most of the current CAR approaches have relied on the use of surface antigens expressed on normal cells and not cancer-specific. CD19 is the most explored antigen so far for targeting CAR-T cells against B cell hematologic malignancies, followed by CD20, B cell maturation antigen (BCMA) and others (14). The lack of specificity can lead to unwanted depletion of normal cell populations, which is a general concern in the field. In addition, overstimulation CAR-T cells *in vivo* due to abundant antigen existence can lead to severe immune-toxicities (15).

On the other hand, tumors associated with viral infections open the prospect of exploring viral antigens not expressed on the surface of normal cells for CAR-T cell engineering. The gp350 envelope protein is abundantly expressed on infected cells during EBV lytic reactivation and sporadically on the surface of latently infected cells, representing a potential virus-specific therapeutic target for CAR-T cells. Our current goal was to advance towards the clinical development of gp350CAR-T cells to treat patients with different types of EBV-associated malignancies.

Therefore, we tested several novel gp350CAR designs in LVs. SIN-LVs were used due to their ability and to efficiently transduce both dividing and non-dividing cells and to stably integrate in genomic locations not likely to be harmful for insertional mutagenesis. Further, GMP production of 3^rd^ generation LVs (four plasmids transient transfection system) is well established in academic centers and companies. LV clinical utilization and boomed in recent years with several optimizations and tests confirming their consistent and broad usability in the immune and gene therapy fields leading to several approved cell therapies (16).

Among the four gp350CAR-T designs tested here, the LV 7A1-gp350CAR with the Fc Hinge and the CD28.CD3ζ signaling domain showed superior performance for reactivity and killing of gp350^+^ targets. These results corroborate our previous studies showing higher performance of 7A1-gp350-CD28.CD3ζ-CAR T cells generated after retroviral vector transduction compared to other RV designs with the 6G4 scFv (9) or incorporating 4-1BB.CD3ζ (Stripecke et al, unpublished data). The Sadelain group extensively compared CD28.CD3ζ and 4-1BB.CD3ζ co-stimulation designs in stress tests using the CD19CAR-T cells in the Nalm-6 leukemia xenograft model (17). They showed that CAR-T cells incorporating CD28.CD3ζ and infused at low doses (1x10^5^ - 2x10^5^) produced significantly higher tumoricidal effects against leukemia development than CAR-T cells with 4-1BB.CD3ζ co-stimulation (17). Since EBV has evolved several mechanisms of immune evasion, we speculate that the CD28.CD3ζ signaling may provide superior potency for the T cells to subvert the EBV immune suppressive signals.

Using scale-up GMP methods, we therefore produced and purified the selected LV incorporating 7A1-gp350-CD28.CD3ζ-CAR and reached consistent high titers (range of 1x10^8^ TU/ml) and purity characteristics. The GMP-grade purified and high-titer ZT002 vector enabled a straightforward upscaling of gp350CAR-T production, and the runs of the cell product showed high purity, viability and sterility. Our CAR-T cell manufacturing process was relatively simple, as the T cells were not enriched prior to transduction, the MOI was low (1), and after transduction the T cells could be cultivated for nine to ten days in the presence of generic clinical-grade IL-2. Three consecutive cell production batches resulted in >80% CAR^+^, approximately 100-fold expansion, range of 4x10^9^ total viable T cells, similar rations of CD8^+^ and CD4^+^ T cells and approximately two vector copies per genome. These results are comparable to CAR-T cells transduced with other lentiviral vectors (18, 19).

The GMP-like gp350CAR-T cells showed *in vitro* cytotoxic potency against five different types of gp350^+^ cell lines as targets (lymphoma, NPC, GC). *In vivo*, gp350CAR-T cells showed a dose dependent incremental therapeutic effect against C6661-1/gp350 NPC growth, but all doses where therapeutic. The abrogation of NPC growth was associated with detectable CAR copies in several tissues and higher infiltration of T cells in tumors. The EBV^+^ NPC model is highly relevant since it broadens the utility of gp350CAR-T cells against solid tumors.

These results underscore the forthcoming advance of gp350CAR-T cells for clinical studies for treatment of patients with EBV^+^ gp350-positive NPCs associated with EBV lytic infections. Some specialized clinical centers are able to produce adoptive virus-specific T cells (VSTs) to treat EBV^+^ LPD, lymphomas as well as nasopharyngeal cancers (20–22). Autologous or third party VSTs are stimulated *ex vivo* with viral latent antigens (such as EBNA1 and LMP2) and cell lots passing batch release are infused, resulting in overall beneficial clinical responses (23). However, for their generation, VSTs strongly rely on viral epitopes presented by the tumors *via* the human leukocyte antigen type I (HLA-I) for their recognition by cognate T cell receptors (TCRs) and their destruction. Nevertheless, EBV is known to down-regulate HLA-I, which may negative impact on the function of VSTs, whereas CAR-T cells are HLA-independent.

The uses of CAR-T cells are expanding beyond oncology and have already been validated against different infectious disease such as EBV (9, 24, 25), human cytomegalovirus (HCMV) (26), human immune deficiency (HIV) (27), and hepatitis C virus (HCV) (28). Therefore, an exciting and highly dynamic “synthetic biology” antiviral CAR-T field is evolving. Taken together, our data confirmed our initial proof-of-concept antitumor effects of gp350CAR-T cells, advanced the cell manufacturing to the GMP level, and the next goal is to evaluate their clinical potential against NPC.

## Concluding remarks

- CAR-T cells targeting the EBV lytic antigen gp350 could be produced after lentiviral vector transduction using GMP-like methods and in sufficient numbers for clinical uses.
- gp350CAR-T cells recognized and killed several cell lines expressing gp350.
- A xenograft mouse model of NPC confirmed the *in vivo* potency of gp350CAR-T cells.

## Data availability statement

The raw data supporting the conclusions of this article will be made available by the authors, without undue reservation.

## Ethics statement

The studies involving human participants were reviewed and approved by Shanghai Zhaxin Traditional Chinese and Western Medicine hospital (study protocol number: LP202006). The patients/participants provided their written informed consent to participate in this study. The animal study was reviewed and approved by research ethics committee at Guangzhou Regenerative Medicine and Health, Guangdong Laboratory (GRMH-GDL) IACUC serial number: 2020125.

## Author contributions

XZhang provided overall conceptualization and project leadership, wrote and the first draft of the manuscript and revised the final version. XZhang, TW, YL, ML, ZH developed technologies and assays, performed *in vitro* experiments and analyzed data. XZhu performed animal studies. DH provided funding, administered resources and provided project leadership. LZ, YW and LL provided NPC patient tissue samples. FK revised or conducted the statistical analyses. RS provided overall conceptualization supervised the data analyses and revised the final manuscript. All authors contributed to the article and approved the submitted version.
