# Supplementary material for: GMP development and preclinical validation of CAR-T cells targeting a lytic EBV antigen for therapy of EBV-associated malignancies
Source: Front Immunol. 2023 Feb 2;14:1103695. doi: 10.3389/fimmu.2023.1103695 (PMC9932894; doi:10.3389/fimmu.2023.1103695)

**Table S1. List of antibodies and purified proteins used in the study.**

| **gp350 staining** | | | | |
| --- | --- | --- | --- | --- |
| **Antibody** | **Fluorochorome conjugate** | **Clone/**  **Dilution** | **Company or Provider** | **Order Number** |
| Rat anti-gp350 monoclonal  Mouse anti-rat IgG | N/A  AF647 | 7A1  1:200  Polyclonal  1:800 | Kindly provided by Prof. Zeidler Munich, Germany  Jackson ImmunoResearch Laboratories | N/A  212-605-082 |
| Mouse anti-gp350 monoclonal  Goat anti-mouse IgG H&L (pre-absorbed) | N/A  Alexa Fluor 488 | 72A1  1:200  Polyclonal  1:800 | Merck Millipore  Jackson ImmunoResearch Laboratories | MAB10219  115-546-062 |
| **Detection of T cells** | | | | |
| **Antibody** | **Fluorochorome conjugate** | **Clone/**  **Dilution** | **Company** | **Order Number** |
| Mouse anti-human CD3 monoclonal | APC | [HIT3a](https://www.biolegend.com/en-us/search-results?Clone=HIT3a)  1:100 | Biolegend | 300312 |
| Mouse anti-human CD3 monoclonal | PE | [HIT3a](https://www.biolegend.com/en-us/search-results?Clone=HIT3a)  1:100 | Biolegend | 300308 |
| Mouse anti-human CD4 monoclonal | BV650 | SK3  1:100 | BD Biosciences | 563875 |
| Mouse anti-human CD8 monoclonal | PE-CY7 | SK1  1:100 | Biolegend | 344712 |
| **Detection of CAR** | | | | |
| **Protein/**  **Antibody** | **Fluorochorome conjugate** | **Clone/**  **Dilution** | **Company** | **Order Number** |
| Goat Anti-Human IgG, Fcγ fragment specific | AF 647 | Polyclonal  1:200 | Jackson ImmunoResearch Laboratories | 109-606-170 |
| EBV gp350 Protein (His Tag)  anti-His Tag Antibody | N/A  PE | N/A  1:25  [J095G46](https://www.biolegend.com/en-us/search-results?Clone=J095G46)  1:50 | Sino Biological  Biolegend | 40373-V08B  362603 |
| **Immunohistochemistry analysis** | | | | |
| **Antibody** | **Fluorochorome conjugate** | **Clone/**  **Dilution** | **Company Company or Provider** | **Order Number** |
| Mouse hybridoma | N/A | OT6  1:200 | Provided by Prof. Jaap Middeldorp, Amsterdam University Medical Center and manufactured by Helmholtz Zentrum Munich, Germany |  |
| Anti-CD3D & CD3E Heterodimer Antibody | N/A | #301  1:200 | Sino Biological | CT026-R301 |

**Table S2. Titer measurement of ZT002 lentiviral vector generated after different production scales.**

|  | 48h supernatant | |
| --- | --- | --- |
|  | Physical titer  (P24 - pg/ml) | Activity titer  (Jurkat - TU/ml) |
| 50 ml Flask | 1.39 x 10^6^ | 6.54 x 10^7^ |
| 500 ml Flask | 3.29 x 10^6^ | 2.59 x 10^7^ |
| 5L WAVE bioreactor | 1.18 x 10^6^ | 4.50x 10^7^ |

**Table S3. Batch release criteria of LV-ZT002.**

| **Items** | **Release criteria** |
| --- | --- |
| **Appearance** | Colorless or light yellowish solution |
| **Sequencing analysis** | Sequence 100% identical |
| **pH** | 6.5~7.5 |
| **Osmosis** | 260~335 mOsm/kg |
| **Visible particles** | Not detected |
| **SV40LTA residue** | ≤1.0×10^7^ copies/ml |
| **E1A residue** | ≤1.0×10^7^ copies/ml |
| **Host DNA residue** | ≤1000 ng/mL |
| **Plasmid DNA residue** | ≤1.0×10^10^ copies/ml |
| **Host protein residue** | ≤2000 ng/ml |
| **Nuclease residue** | ≤1 ng/ml |
| **Bacterial endotoxin** | ≤10 EU/ml |
| **Sterility** | Sterile |
| **Mycoplasma** | Negative |
| **RCL** | Negative |
| **Physical titer** | ≥1.0×10^6^ pg/ml |
| **Activity titer** | ≥1.0×10^7^ TU/ml |

**Table S4. Batch release criteria of gp350CAR-T cells.**

| Item | Methodology | Sample collection point | Release Criteria |
| --- | --- | --- | --- |
| Characteristics | observation | Before/after recovery of final product | White or light yellowish |
| Percentage of viable cells | NC-200 cell counter | Recovered cells | ≥ 70% |
| gp350CAR expression | Flow cytometric analysis | Recovered cells | CD3^+^ CAR^+^ ≥ 20% |
| Percentage of CD3+ cells | Flow cytometric analysis | Recovered cells | CD3^+^ ≥ 80% |
| Percentage of irrelevant cells | Flow cytometric analysis | Recovered cells | CD19^+^CD45^+^≤5% |
| Bacteria endotoxin | Gel method | Recovered cells | < 0.25 EU/mL |
| IFN-γ | ELISA | Recovered cells | ≥ 2 (test sample/ control sample) |
|  |  |  |  |
| Sterility | Membrane filtration  （pharmacopeia） | Final product | Negative |
| Mycoplasma | Culture method DNA Fluorescence（pharmacopeia） | Final product | Negative |
| Osmosis | Cryoscopic method  （pharmacopeia） | Recovered final product | 1000-1500 mOmsm/kgH2O  (Pending) |
| PH | PH meter  （pharmacopeia） | Recovered final product | 7-8 |
| Copy number per cell | qPCR | Recovered final product | ≤ 5 copies/cell |
| RCL | qPCR | Recovered cells | (Pending) |
| IL-2 residue | ELISA | Supernatant of recovered final product | (Pending) |
| Magnetic beads residue | Microscopic observation | Cell suspension after recovery | No residues |

**Table S5. Semi-quantitative microscopic assessment of cell frequencies and expression levels of CD3 and gp350 in C666.1/gp350 tumor tissue sections obtained from mice treated with saline, Mock-T cells or gp350CAR-T cells. Cells with strong and moderate staining levels were quantified separately.**

| **Figures** | **Intervention** | **CD3 staining** | **Gp350 staining** |
| --- | --- | --- | --- |
| Figure 5B  (Case Number 1) | Saline | negative | **30%+++**  30%++  Average: 30% |
|  | Mock-T | ~1% cells +++ | **50%+++**  30%++  Average: 40% |
|  | gp350CAR-T | ~2% cells +++ | **35%+++**  25%++  Average: 30% |
| Figure S2 (Case Number 2) | Saline | negative | **45%+++**  35%++  Average: 40% |
|  | Mock-T | <1%++ | **40%+++**  35%++  Average: 37.5% |
|  | gp350CAR-T | ~3%+++ | **40%+++**  30%++  Average: 35% |
| Figure S2 (Case Number 3) | Saline | negative | **45%+++**  40%++  Average: 42.5% |
|  | Mock-T | <1%++ | **60%+++**  30%++  Average: 45% |
|  | gp350CAR-T | ~2%+++ | **20%+++**  40%++  Average: 30% |

+++, strong staining; ++, moderate staining

**Figure S1:** **Additional *in vitro* cytotoxicity analyses for comparison of four anti-gp350 CAR-T cells at several E.T ratios (related to Figure 1E).** Lactose dehydrogenase (LDH)-based cytotoxicity assay (16 hours culturing at E:T ratios of 1:2, 1:1, 2:1, 4:1) was used to assess the cytotoxicity of four anti-gp350 CAR-T cells against gp350-positive human oropharyngeal cancer cell lines PCI-1 (PCI-1/gp350). Non-transduced T (Mock-T) cells were included as a negative control. These results are presented as means from two independent donors ± s.d.


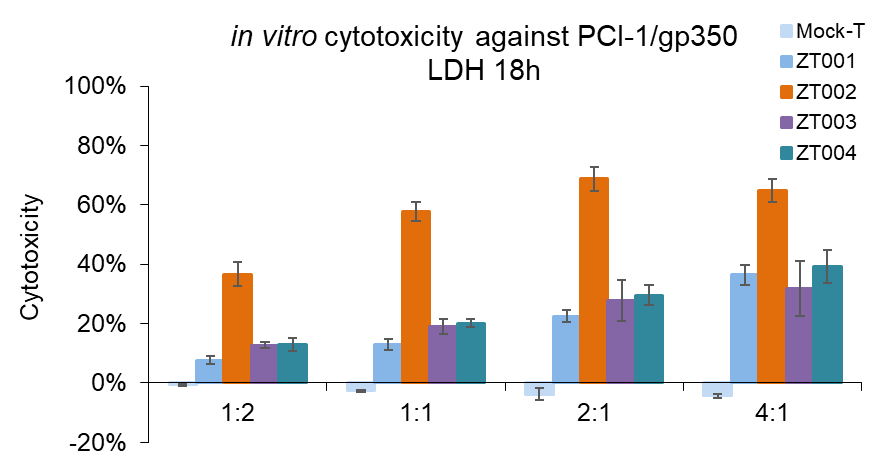


**Figure S2: Correlation between accumulation of gp350CAR-T cells in tumors and loss of gp350^+^ tumor cells (related to Figure 5B and Supplemental Table 5).** (A) Terminal analyses for localization of CD3^+^ T cells and (B) gp350^+^ cells in tumors after IHC staining. Representative samples. Scale bar (black) depicts 50 μm.


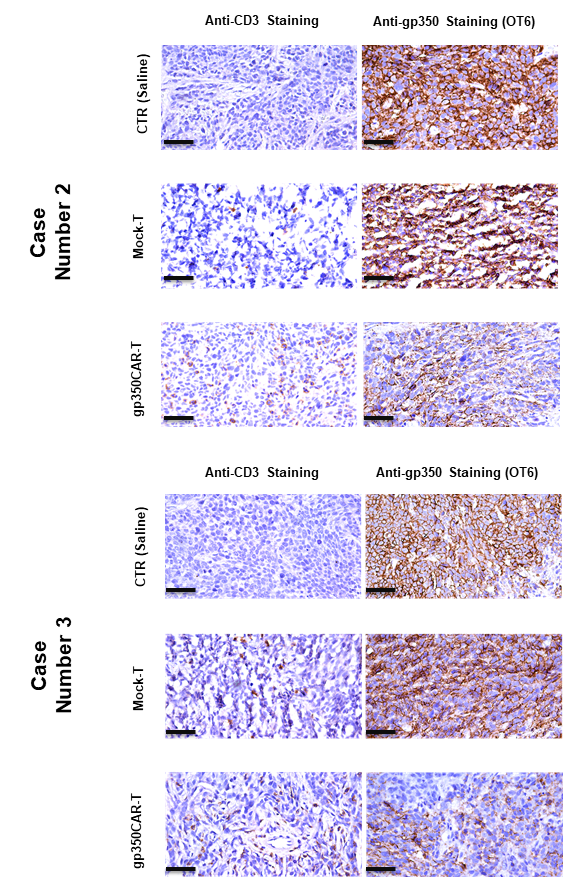


**Figure S3: Pharmacodynamics study performed under good laboratory practices (GLP) to evaluate the effects of the test article in the anti-tumor efficacy in mice (related to Figure 4).** (A) Schematic representation of *in vivo* experiment. NOD-Cg.Prkdc^SCID^IL-2rg^tm1sug^/JicCrl (NOG) mice were injected with 3.21x10^6^ NPC EBV^+^C666.1/gp350 cells s.c. Six days later, mice were injected with vehicle (cryopreservation medium), mock-T or CAR-T cells at escalating cell doses (2x10^6^ CAR-T/mouse, 1x10^7^ CAR-T/mouse, 2x10^7^ CAR-T/mouse, equals to 4.3*10^6^ total T cells/mouse, 2.1x10^7^ total T and 4.3x10^7^ total T cells/mouse respectively) with 10 mice per group. Longitudinal analyses were performed to follow tumor growth and body weight until Day 42 post T cell infusion. (B) Longitudinal analyses of tumor volume (mm^3^). On D42, the tumor volumes of mice in vehicle group and Mock-T group increased to 718.86±115.56 mm^3^ and 560.07±84.81 mm^3^, respectively. The final tumor volumes of mice in the low dose, medium dose and high dose of test article groups were 334.30±125.11 mm^3^, 268.13±111.95 mm^3^ and 228.02±96.79 mm^3^, respectively. The tumor volumes of mice in test article groups were significantly smaller than those of mice in the vehicle group and T cell control group (*P*<0.001). The test article inhibited tumor cell proliferation in a dose-dependent manner. (C) Body weights measurements. On D42, mice in the T cell control group had significantly lower body weights compared with the vehicle group (16.8±2.5 g vs 20.8±1.4 g, *P*<0.001), which was considered to be associated with Graft-versus-Host Disease (GvHD). The final weights of mice in test article groups were 20.9±0.8 g, 21.0±2.0 g and 19.7±2.3 g, respectively. Compared with the vehicle group, test article had no significant effect on the body weights of mice *(P*>0.05). The study was conducted by JOINN Laboratories (Suzhou) Co. Ltd. (Jiangsu, China).


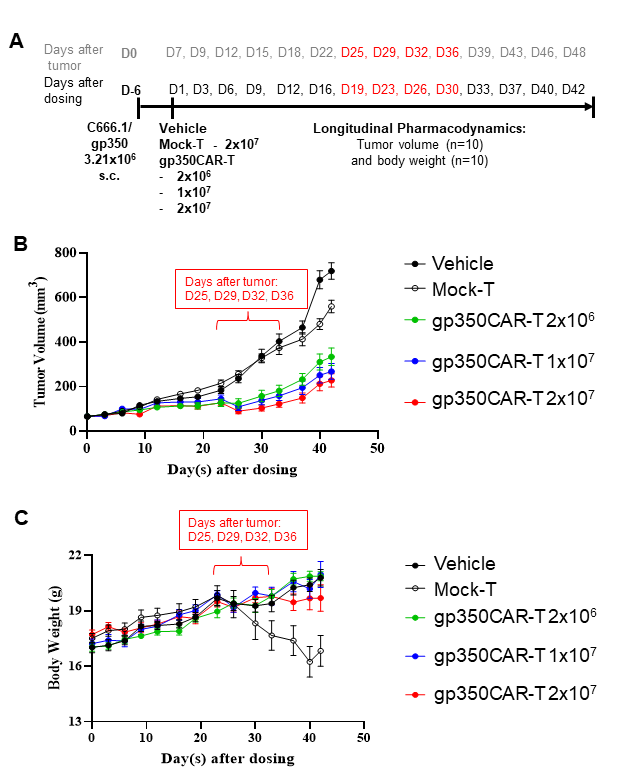


**Figure S4: Pharmacokinetics study performed under good laboratory practices (GLP) to evaluate the bio-distribution of the test article in mice (related to Figures 4 and 5).** (A) Schematic representation of *in vivo* experiment. NOD-Cg.Prkdc^SCID^IL-2rg^tm1sug^/JicCrl (NOG) female and male mice were injected with 3x10^6^ NPC EBV^+^C666.1/gp350 cells s.c. The study was designed with a single dose of 2×10^7^ CAR-T cells/mouse. 0.2 mL of test article with cell density of 1.0×10^8^ CAR-T cells/mL was injected i.v. into each mouse on D1. All surviving animals were euthanized at 24h (D2), 144h (D7), 312h (D14), 648h (D28),1152h (D49) and 1656h (D70) after dosing with 5 mice/sex each time, and tissues and whole blood samples were collected at the same time point. The CAR gene DNA copies of test article in the whole blood and tissues after dosing were determined using a validated qPCR method, and the lower limit of quantification was 25 copies/μL (50 copies/reaction). (B) Distribution of CAR gene DNA in the tissues of tumor-bearing NOG female mice. (C) Distribution of gp350CAR DNA in the tissues of tumor-bearing NOG male mice. A certain number of the CAR gene DNA copies could be detected in the whole blood and tissues of male and female mice. The tumor tissues of female and male mice peaked on D7 and D14, respectively, after dosing and then gradually decreased over time to the end of observation. The copies of CAR gene DNA in the tissues gonads of male and female mice and the lung tissues of males gradually increased over time after dosing to the end of observation (1656 h after dosing). The copies of other tissues gradually decreased over time after the first peak and then reached the second peak on D49 after dosing. PCR signals of the gp350CAR-T cells were highly detectable in blood-rich tissues such as the tumor, whole blood, lung, spleen, liver and kidney, and immune organ tissues such as the spleen and mesenteric lymph nodes. Among the analyzed tissues, the detection was highest in the tumor tissues. The study was conducted by JOINN Laboratories (Suzhou) Co. Ltd. (Jiangsu, China).


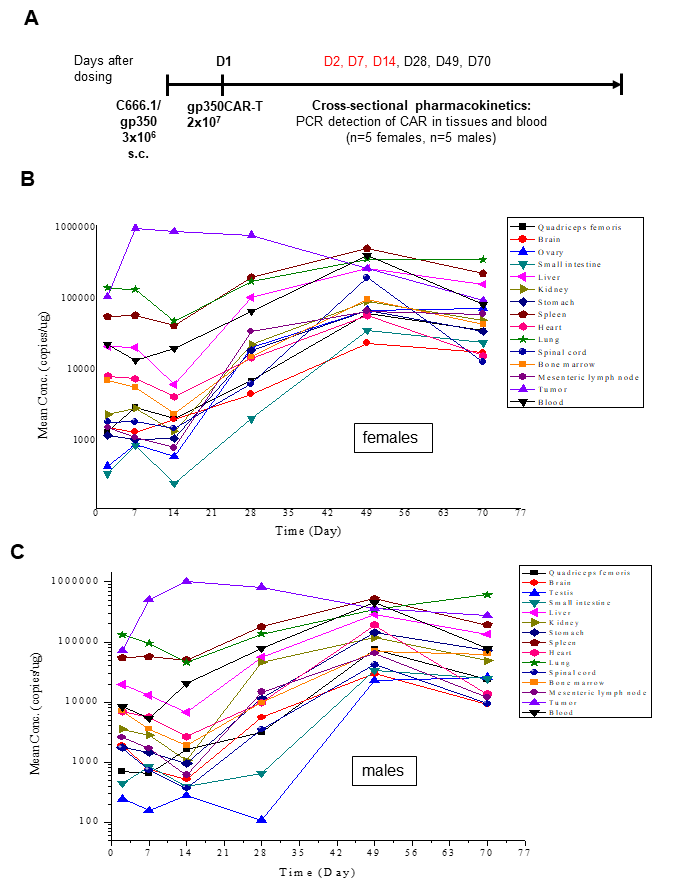

Supplement: Supplementary file 1 [file DataSheet_1.docx]
